# Meningococcal carriage in children and young adults in the Philippines: a single group, cross-sectional study

**DOI:** 10.1017/S0950268816002119

**Published:** 2016-09-22

**Authors:** M. L. A. GONZALES, V. BIANCO, A. VYSE

**Affiliations:** 1 Department of Pediatrics, College of Medicine, University of the Philippines Manila, Manila, Philippines; 2 GSK Vaccines, Wavre, Belgium

**Keywords:** Epidemiology, *Neisseria meningitidis*

## Abstract

This cross-sectional prevalence study investigates meningococcal carriage for the first time in a Southeast Asian population. Posterior pharyngeal swabs were collected between August 2013 and March 2014 from 937 healthy Filipinos aged 5–24 years attending school or university in Manila. Of these, 35 were found to be carriers giving an overall carriage prevalence of 3·7% [95% confidence interval (CI) 2·6–5·2]. Carriage was associated with age (*P* < 0·001) and was highest (9·0%, 95% CI 5·5–13·8) in subjects aged 10–14 years, but was comparatively low (<3%) in all other age groups considered. This suggests that an immunization programme in the Philippines designed to reduce carriage acquisition and induce herd immunity may require a vaccine dose before the age of 10 years. Serogroup B was most commonly carried (65·7%, 95% CI 47·8–80·9), with a small number of carriers for serogroups C, Y and W also present. Two individuals (5·7%, 95% CI 0·7–19·2) who were simultaneously carrying multiple serogroups were identified. This exploratory study provides valuable insight into the asymptomatic carriage of *Neisseria meningitidis* in a healthy subset of the Filipino population and illustrates the importance of generating local carriage data.

## INTRODUCTION

While cases of invasive meningococcal disease reflect the most important outcome in terms of public health, they play only a limited role in transmission, most of which occurs silently via asymptomatic carriage [1]. It is now well accepted that meningococcal carriage is relatively frequent and varies considerably with age. The highest prevalence is often found in adolescents and young adults suggesting that this age group plays a key role in transmission [2]. However, the dynamics and epidemiology of meningococcal carriage remain to be fully understood, with existing data often relatively limited [2–4]. Age-stratified carriage profiles may vary to some extent in different population groups due to different cultural and social demographics, which likely influence transmission patterns and risk factors for carriage acquisition [5, 6]. While meningococcal carriage has now been investigated in some detail in the African meningitis belt [7], an understanding of how carriage prevalence varies by capsular serogroup in other global regions remains limited at present, with existing data suggesting that while serogroup B often dominates, serogroups C, Y and W are also frequently carried [3]. Furthermore, carriage isolates may also often lack capsule expression [8, 9].

Meningococcal vaccines that have the ability to prevent acquisition of carriage and thus reduce transmission can provide important indirect protection and induce herd immunity [8, 10]. Therefore, if key age groups carrying and transmitting meningococcus are included in meningococcal vaccination programmes, cost-effectiveness and long-term control of the disease can be optimized. Such ability to induce herd immunity is considered a class effect for meningococcal capsular polysaccharide conjugate vaccines [11–13]. However, it is yet to be demonstrated for the new protein-based meningococcal vaccines targeting serogroup B that are now available, although it is considered to be a potential property they may possess [9, 14]. To maximize the impact on carriage and any indirect protection obtained, a better understanding of how carriage varies by age in specific population groups is therefore important for informing vaccination strategy by identifying candidate age groups particularly involved with transmission for potential inclusion in vaccination programmes [15].

Data describing the epidemiology of meningococcus in Southeast Asia are currently very limited. At present, countries in this region do not undertake routine surveillance for invasive meningococcal disease, with cases rarely identified and confirmed. The extent of the disease burden is therefore poorly understood, and no country in the region currently includes meningococcal vaccines in its routine immunization schedule [16]. Furthermore, while the majority of published meningococcal carriage studies to date have been undertaken in Europe [2, 3] and carriage data are now starting to emerge from the African meningitis belt [7] and both Latin and North America [8, 17], so far no meningococcal carriage studies have been undertaken in populations from Southeast Asia. The purpose of this study is to start addressing this knowledge gap by investigating asymptomatic carriage in healthy Filipinos aged 5–24 years. Published data describing the epidemiology of meningococcal disease in the Philippines are currently very limited. However, a large outbreak of meningococcal disease attributed to serogroup A was reported to have occurred at the beginning of the 21st century [16], suggesting that the Philippines could be an interesting candidate country in Southeast Asia in which to undertake a carriage study.

## METHODS

This observational cross-sectional meningococcal carriage prevalence study was performed using posterior pharyngeal swabs [18] collected between August 2013 and March 2014 from healthy Filipinos aged 5–24 years.

Participants were volunteers recruited from three sites in Manila: one elementary school, one high school and one university. A stratified random sampling method using grade/school year level as stratum was used. About 75 subjects were enrolled per grade/school year level in order to achieve a target sample size of 1000 subjects divided into a 1:1:1:1 ratio of ~250 subjects from each of the following age groups: 5–9, 10–14, 15–19 and 20–24 years. The swabbing procedure was undertaken at the University of the Philippines Manila – Philippines General Hospital (UPM-PGH). One posterior pharyngeal swab was collected per subject. Participants were also asked to complete a questionnaire to identify age, gender, academic institution attended and possible risk factors for carriage (including smoking status, type of accommodation, household size and number of siblings). Participants with medical conditions or those taking any medication considered to affect the likelihood of meningococcal carriage or the ability to obtain a suitable posterior pharyngeal swab were excluded. This included administration of antibiotics at any stage during the 2 weeks prior to swabbing, and evidence of previous vaccination with a meningococcal conjugate vaccine.

Posterior pharyngeal swabs were collected by swabbing first the tonsils then the posterior pharyngeal wall behind the uvula through the mouth using sterile polyester-tipped swabs and immediately plated onto modified Thayer–Martin agar media. These were incubated at 35 °C (in 3–7% CO_2_) using standard microbiological techniques in the microbiology laboratory at UPM-PGH. Culture plates were examined at 24 h and 72 h after inoculation for suspected colonies of *Neisseria meningitidis* (smooth, round moist grey to white colonies) and a Gram stain was performed to confirm the presence of Gram-negative diplococci. Colonies were subcultured for isolation; with candidate pure *Neisseria* cultures identified using analytical profile index identification. Candidate colonies were stored using Microbank vials at −80 °C and subsequently transferred under dry ice to a further laboratory (BARC-SA, Bio Analytical Research Corporation, South Africa) for confirmation as *N. meningitidis* using a multiplex polymerase chain reaction (PCR) kit (Multiplex PCR-FTD Bacterial meningitis; Fast Track Diagnostics Ltd, Luxembourg; www.fast-trackdiagnostics.com). A subsequent PCR test was then used for serogrouping [19], with further characterization undertaken using multilocus sequence typing (MLST) [20].

Statistical analyses were performed using SAS software version 9.2 (SAS Institute Inc., USA). Results are presented descriptively as frequencies and proportions of posterior pharyngeal swab samples where *N. meningitidis* was detected, with corresponding exact 95% confidence intervals (CI). Exploratory analyses assessed the associations between candidate risk factors and meningococcal carriage using Fisher's exact test.

### Ethical statement

All procedures used in this study complied with the ethical standards of the relevant institutional committee on human experimentation (University of the Philippines Manila Research Ethics Board) and with the Helsinki Declaration of 1975, as revised in 2008.

## RESULTS

Posterior pharyngeal swabs were obtained from a total of 937 subjects aged 5–24 years. Table 1 summarizes the demographics of the study population. Culture identified 48 individuals as possibly carrying meningococci. Of these, 35 were subsequently confirmed by PCR, giving an overall carriage prevalence of 3·7% (95% CI 2·6–5·2). Further characterization by serogroup and clonal complex is shown in Table 2 and the carriage prevalence stratified by both age group and serogroup is shown in Table 3. A single serogroup only was isolated from 28 individuals, with 23 carrying serogroup B (65·7%, 95% CI 47·8–80·9), three carrying serogroup C (8·6%, 95% CI 1·8–23·1) and two carrying serogroup Y (5·7%, 95% CI 0·7–19·2). Two individuals (5·7%, 95% CI 0·7–19·2) were found to be simultaneously carrying multiple serogroups (B/Y/W and Y/W, respectively). Capsular serogroup was not identified for five (14·3%, 95% CI 4·8–30·3) carriers. In the 35 carriage isolates identified in this study, clonal complex was identified for 31 isolates. ST175C (nine carriers), ST41/44C/L3 (seven carriers) and ST5656 (three carriers) were the most frequently identified.

**Table 1.** Study population stratified by demographic characteristics and *Neisseria meningitidis* carriage status (as defined by multiplex PCR result)

Demographic characteristic/category	Non-carrier	Carrier	Total
	(*N* = 902)	(*N* = 35)	(*N* = 937)
	*n* (%)	*n* (%)	*n* (%)
Age group (years)			
5–9	242 (26·8)	6 (17·1)	248 (26·5)
10–14	191 (21·2)	19 (54·3)	210 (22·4)
15–19	226 (25·1)	6 (17·1)	232 (24·8)
20–24	243 (26·9)	4 (11·4)	247 (26·4)
Gender			
Female	512 (56·8)	20 (57·1)	532 (56·8)
Male	390 (43·2)	15 (42·9)	405 (43·2)
Smoking status			
Smoker	22 (2·4)	2 (5·7)	24 (2·6)
Non-smoker	880 (97·6)	33 (94·3)	913 (97·4)
Educational institute attended			
University	391 (43·3)	7 (20·0)	398 (42·5)
School	511 (56·7)	28 (80·0)	539 (57·5)
Type of accommodation			
Flat or apartment	291 (32·3)	7 (20·0)	298 (31·8)
House	503 (55·8)	23 (65·7)	526 (56·1)
Other	108 (12·0)	5 (14·3)	113 (12·1)
Number of people sharing household			
Live alone	28 (3·1)	1 (2·9)	29 (3·1)
1–4	331 (36·7)	10 (28·6)	341 (36·4)
5–9	411 (45·6)	14 (40·0)	425 (45·4)
⩾10	132 (14·6)	10 (28·6)	142 (15·2)
Number of siblings			
0	55 (6·1)	0 (0·0)	55 (5·9)
1	158 (17·5)	3 (8·6)	161 (17·2)
2	258 (28·6)	7 (20·0)	265 (28·3)
3	201 (22·3)	11 (31·4)	212 (22·6)
⩾4	230 (25·5)	14 (40·0)	244 (26·0)

PCR, Polymerase chain reaction.

**Table 2.** Individual listing of the 35 *Neisseria meningitidis* isolates showing serogroup and clonal complex (MLST results)

No.	Serogroup	MLST
1	B	Indeterminate
2	B	ST334C
3	B	ST8683
4	Unassigned	ST175C
5	B	ST3013
6	B	ST5617
7	B	ST41/44C/L3
8	B	ST334C
9	B/Y/W	ST11C/ET37C
10	Unassigned	ST175C
11	B	ST41/44C/L3
12	B	ST4690
13	Y/W	ST11C/ET37C
14	B	ST41/44C/L3
15	B	ST5656
16	B	Indeterminate
17	Unassigned	ST175C
18	C	ST5656
19	B	ST41/44C/L3
20	Unassigned	ST175C
21	Y	ST175C
22	B	ST41/44C/L3
23	C	ST175C
24	B	ST5656
25	B	Indeterminate
26	B	ST41/44C/L3
27	B	Indeterminate
28	B	ST41/44C/L3
29	B	ST5617
30	Y	ST175C
31	Unassigned	ST175C
32	B	ST53C or ST1117C
33	B	ST175C
34	B	ST32C/ET5C
35	C	ST2954

MLST, Multilocus sequence typing; unassigned, no detectable serogroup.

**Table 3.** *Neisseria meningitidis* carriers stratified by age and serogroup

Age group (years)	Serogroup	*N*	% (95% CI)
5–9 (*N* = 6)	B	5	83·3 (35·9–99·6)
	Unassigned	1	16·7 (0·4–64·1)
10–14 (*N* = 19)	B	11	57·9 (33·5–79·7)
	B/Y/W	1	5·3 (0·1–26·0)
	C	2	10·5 (1·3–33·1)
	Unassigned	3	15·8 (3·4–39·6)
	Y	1	5·3 (0·1–26·0)
	Y/W	1	5·3 (0·1–26·0)
15–19 (*N* = 6)	B	4	66·7 (22·3–95·7)
	Unassigned	1	16·7 (0·4–64·1)
	Y	1	16·7 (0·4–64·1)
20–24 (*N* = 4)	B	3	75·0 (19·4–99·4)
	C	1	25·0 (0·6–80·6)
5–24 (*N* = 35)	B	23	65·7 (47·8–80·9)
	B/Y/W	1	2·9 (0·1–14·9)
	C	3	8·6 (1·8–23·1)
	Unassigned	5	14·3 (4·8–30·3)
	Y	2	5·7 (0·7–19·2)
	Y/W	1	2·9 (0·1–14·9)

CI, Confidence interval; unassigned, no detectable serogroup.

Carriage prevalence varied by age group and was highest in participants aged 10–14 years (9·0%, 95% CI 5·5–13·8, *P* < 0·001). There was no evidence that carriage was associated with gender (*P* > 0·1) or with the type of living accommodation (*P* > 0·1). There was also no evidence that being a current smoker was associated with carriage (*P* > 0·1), although the majority (>97%) of the study population were non-smokers. A higher carriage prevalence was observed in subjects with more siblings, and also in those who shared a household with a larger number of inhabitants. However, while there was weak evidence (*P* = 0·08) to support an association of carriage in individuals with more siblings, there was no statistical evidence supporting an association of carriage with a larger number of household inhabitants (*P* > 0·1).

## DISCUSSION

This is the first study investigating meningococcal carriage in a Southeast Asian population. Its main purpose was to be exploratory and look for evidence of carriage, rather than attempting to definitively describe meningococcal carriage in the region. It therefore did not study the full age range, but rather focused on individuals aged 5–24 years given that published data suggest the likelihood of finding carriers might be highest in this age group [2, 3]. The study was also restricted to a local Filipino population recruited from Manila, so caution should be exercised when extending the findings to other Southeast Asian populations. An additional limitation when undertaking meningococcal carriage studies is that there is currently no standardized or optimized methodology for sampling, and subsequently detecting and identifying any carried meningococci [3, 8, 21, 22]. Methodological approaches can therefore differ to some extent in studies, suggesting that comparison of findings between different carriage studies should be undertaken with some caution.

The overall carriage prevalence detected was relatively low (3·7%), but indicates that meningococcus is present and circulating in this subset of the Filipino population. As expected, carriage prevalence varied by age group but was found to be highest in those aged 10–14 years. This contrasts with the findings of a meta-analysis of existing (mainly European) carriage data, which highlighted that while carriage prevalence often varied substantially between individual studies, highest prevalence was generally observed in older adolescents and young adults [2]. Our findings more closely reflect recent data from the African meningitis belt, where overall carriage prevalence reported was similar and also found to be highest in younger age groups [7]. This suggests that the extent of meningococcal carriage and the age groups most likely to be carriers may vary between different population groups, and is likely to be influenced by local social demographics and cultural behaviours. These data suggest that individuals aged 10–14 years may play a key role in meningococcal transmission in the Philippines. This implies that if a public health need for routine meningococcal vaccination in the Philippines becomes manifest (and potentially also in other Southeast Asian countries), it may be appropriate to consider vaccination of pre-adolescent children prior to entering high school to maximize any indirect protection and enable optimal control of the disease.

The majority of carried meningococci identified in this study had a serogroup B polysaccharide capsule. Carriage of serogroup C, Y, and W meningococci was also observed, but much less frequently. For a small proportion of carriers, the serogroup could not be identified which may reflect the presence of non-capsulated isolates, or those expressing capsular serogroups other than A, B, C, W or Y. These findings are consistent with a review of carriage studies undertaken in Europe, where serogroup B was found to be the dominant serogroupable meningococcus carried [3]. Further characterization by MLST indicated that a considerable proportion of the clonal complexes identified were associated with strains causing invasive disease, with 21/31 (67·7%) clonal complexes identified reflecting those isolated from confirmed cases of invasive meningococcal disease reported through routine surveillance in the European region in 2012 [23]. Given that carriage is considered a prerequisite for disease [24], carriage of such strains known to cause invasive disease therefore indicates a clear potential for meningococcal disease to occur in the Filipino population, particularly due to serogroup B. This is somewhat surprising as existing data primarily indicate the occurrence of serogroup A disease in the Philippines, with a documented outbreak in 2004–2005 [16]. However, it is difficult to assess the burden of invasive meningococcal disease in the Philippines given the current absence of routine surveillance and limited infrastructure to diagnose, confirm and characterize suspected cases. The lack of reported serogroup B disease therefore does not necessarily exclude its occurrence, and these carriage data reinforce the need to introduce surveillance capacity to more accurately assess the disease burden and serogroup distribution both in the Philippines and across the region more widely [16].

Knowledge of meningococcal carriage and transmission dynamics can be particularly important when evaluating the potential wider impact of vaccination against invasive disease, whether assessed in real-life settings or predictively using mathematical modelling. This can provide valuable insights for informing health policy when vaccination strategies are under consideration. However, the epidemiology of carriage is yet to be fully understood with a number of key questions still remaining, one of which being the extent to which co-colonization by different serogroups may occur, with very little data on this topic currently available [3, 25]. While widespread use of meningococcal vaccines with an ability to prevent acquisition of carriage can induce valuable indirect protection, it also holds potential to induce a serogroup replacement event resulting from increased colonization by other serogroups subsequently filling the ecological niche vacated by vaccine-targeted serogroups. This may lead to an increase in disease caused by non-vaccine serogroups. It is possible that such serogroup replacement could be facilitated by the concurrent co-existence of different meningococcal serogroups in the pharynx, where the removal of one as a result of vaccination may confer an advantage for others that are present [25]. While to date there is no evidence that selective vaccination against certain meningococcal serogroups has led to a clinically significant increase in disease caused by non-vaccine serogroups, this remains an important public health concern associated with wide use of meningococcal vaccines [24, 26]. In this study, a small proportion of individuals were found to be simultaneously carrying more than one capsular serogroup. This finding therefore provides some evidence that different meningococcal serogroups do co-exist in the same ecological niche, with possible implications for a vaccine-driven serogroup replacement event.

Smoking is recognized as being a risk factor for meningococcal carriage [6, 17]. While this study found no evidence of such an association very few participants were smokers, preventing us from being able to meaningfully investigate this aspect. The study results also suggest a higher carriage prevalence in individuals with more siblings and in those who shared accommodation with more inhabitants, which is consistent with the notion of close contact between individuals increasing the likelihood of meningococcal carriage. However, any supporting statistical evidence available was limited due to the relatively small number of carriers identified by the study.

In conclusion, this exploratory study provides valuable insight into the asymptomatic carriage of *N. meningitidis* in a healthy subset of the Filipino population and illustrates the importance of generating local carriage data. Carriage was highest in older children and young teenagers, suggesting that this age group may play an important role in meningococcal transmission in the Philippines and that if an immunization programme in the Philippines designed to reduce carriage acquisition and induce herd immunity ever becomes desirable it may require a vaccine dose before the age of 10 years. Serogroup B was the most frequently identified as being carried, with a considerable number of strains present associated with invasive disease. Future carriage studies in the region should consider incorporating larger study populations, reflecting a wider age range, and also investigate carriage in other population groups from Southeast Asia. Southeast Asian countries are densely populated with considerable socioeconomic, geographical and ethnic diversity, suggesting that meningococcal carriage profiles may vary within the region.
